# Can we predict who will benefit most from biologics in severe asthma? A post-hoc analysis of two phase 3 trials

**DOI:** 10.1186/s12931-023-02409-2

**Published:** 2023-05-02

**Authors:** Wenjia Chen, Helen K. Reddel, J Mark FitzGerald, Richard Beasley, Christer Janson, Mohsen Sadatsafavi

**Affiliations:** 1 Saw Swee Hock School of Public Health, National University of Singapore, MD1 - Tahir Foundation Building, 12 Science Drive 2, Singapore, 117549 Singapore; 2 The Woolcock Institute of Medical Research, The University of Sydney, Sydney, Australia; 3 Respiratory Evaluation Sciences Program, Faculty of Pharmaceutical Sciences, the University of British Columbia, Vancouver, Canada; 4 Medical Research Institute of New Zealand, Wellington, New Zealand; 5 Department of Medical Sciences, Uppsala University, Uppsala, Sweden

**Keywords:** Severe asthma, Biologics, Mepolizumab, Prediction, Treatment response

## Abstract

**Background:**

Individualized prediction of treatment response may improve the value proposition of advanced treatment options in severe asthma. This study aimed to investigate the combined capacity of patient characteristics in predicting treatment response to mepolizumab in patients with severe asthma.

**Methods:**

Patient-level data were pooled from two multinational phase 3 trials of mepolizumab in severe eosinophilic asthma. We fitted penalized regression models to quantify reductions in the rate of severe exacerbations and the 5-item Asthma Control Questionnaire (ACQ5) score. The capacity of 15 covariates towards predicting treatment response was quantified by the Gini index (measuring disparities in treatment benefit) as well as observed treatment benefit within the quintiles of predicted treatment benefit.

**Results:**

There was marked variability in the ability of patient characteristics to predict treatment response; covariates explained greater heterogeneity in predicting treatment response to asthma control than to exacerbation frequency (Gini index 0.35 v. 0.24). Key predictors for treatment benefit for severe exacerbations included exacerbation history, blood eosinophil count, baseline ACQ5 score and age, and those for symptom control included blood eosinophil count and presence of nasal polyps. Overall, the average reduction in exacerbations was 0.90/year (95%CI, 0.87‒0.92) and average reduction in ACQ5 score was 0.18 (95% CI, 0.02‒0.35). Among the top 20% of patients for predicted treatment benefit, exacerbations were reduced by 2.23/year (95% CI, 2.03‒2.43) and ACQ5 score were reduced by 0.59 (95% CI, 0.19‒0.98). Among the bottom 20% of patients for predicted treatment benefit, exacerbations were reduced by 0.25/year (95% CI, 0.16‒0.34) and ACQ5 by -0.20 (95% CI, -0.51 to 0.11).

**Conclusion:**

A precision medicine approach based on multiple patient characteristics can guide biologic therapy in severe asthma, especially in identifying patients who will not benefit as much from therapy. Patient characteristics had a greater capacity to predict treatment response to asthma control than to exacerbation.

**Trial registration:**

ClinicalTrials.gov number, NCT01691521 (registered September 24, 2012) and NCT01000506 (registered October 23, 2009).

**Supplementary Information:**

The online version contains supplementary material available at 10.1186/s12931-023-02409-2.

## Background

Approximately 5 to 10% of asthma patients have severe or refractory asthma [1–3]. Patients with severe asthma suffer from substantially higher morbidity, mortality, and comorbidities compared to patients with mild or moderate asthma [4, 5], and contribute to nearly 50% of healthcare spending across all asthma patients [6]. Major components of burden are the ongoing risk of exacerbations and poor asthma control. Thus, the prevention of exacerbations and improvement in asthma control are major targets of treatment in guidelines and critical endpoints in severe asthma clinical trials.

Advanced therapeutic antibodies (biologics) herald a new era of therapy in patients with severe asthma. Randomized controlled trials (RCTs) have demonstrated that omalizumab (anti-IgE antibody), mepolizumab, reslizumab (anti-interleukin-5 antibodies), benralizumab (anti-IL5Rα antibody), dupilumab (anti-IL4Rα antibody) and tezepelumab (anti-cytokine thymic stromal lymphopoietin antibody) have efficacy in reducing severe exacerbations, improving asthma control and lung function [7–12] and, in some cases, reducing the use of oral corticosteroids [11, 13, 14]. Subgroup analysis of early clinical trial data identified individual biomarkers such as blood eosinophil count (BEC) and serum IgE for response to biologics [15, 16], which informed the criteria for regulatory approvals and treatment recommendations. However, to what extent multiple patient characteristics and biomarkers in combination can predict response to biologics has not been rigorously evaluated.

Indeed, in both clinical trials and real-world settings, individual responses to biologics are heterogeneous even among patients who are pre-selected for enhanced response based on relevant biomarkers and exacerbation history. Previous analyses have shown that 15–17% of biologic users show no response [17–19] and 43–69% show partial response [19, 20]. Biologics are expensive, costing roughly $10,000 to $30,000 USD per person-year [21]. Consequently, a considerable proportion of eligible patients lack adequate access to biologics, such as in Singapore where the cost-effectiveness profile is currently beyond the accepted willingness-to-pay threshold for formulary approval [22]. The efficiency and cost-effectiveness of biologic therapy could potentially be improved using a ‘precision medicine’ approach to therapy, i.e., basing treatment choices on predicted response given each patient’s unique characteristics [23].

Combining two international RCTs of mepolizumab in severe asthma, this study aimed to investigate whether salient patient characteristics (including biomarkers) in combination can identify subgroups of patients who may have a better response to therapy. We quantified the heterogeneity of treatment benefit, in terms of reduction in the rate of exacerbations or improvement in symptom control, related to biologic therapy.

## Methods

### Data sources

We pooled individual-level data from two double-blind, placebo-controlled trials of mepolizumab for severe asthma: the Dose Ranging Efficacy And safety with Mepolizumab (DREAM, 2009/11–2011/12), and the Mepolizumab as Adjunctive Therapy in Patients with Severe Asthma (MENSA, 2012/10–2014/01) studies [10, 24].

During the 52-week DREAM study, 621 patients were randomly assigned (in a 1:1:1:1 ratio) to receive one of the three doses of intravenous mepolizumab (75 mg, 250 mg, 750 mg) or matched placebo every 4 weeks. During the 38-week MENSA study, 576 patients were randomized (in a 1:1:1 ratio) to receive placebo, a 75 mg intravenous dose, or a 100 mg subcutaneous dose of mepolizumab, every 4 weeks. Both studies had similar inclusion criteria related to clinical diagnosis, eosinophilic asthma, and lung function, with details provided in *Appendix S1*. In addition, both studies required patients to have a confirmed history of 2 or more asthma exacerbations requiring treatment with oral corticosteroids (OCS) in the preceding 12 months.

### Study design and sample

To ensure consistency and achieve a desirable sample size for minimizing potential model overfitting [25], this study included patients who received placebo or intravenous 75 mg doses of mepolizumab because this regimen was included in both trials. Patients were followed from the date of study enrolment until the end of study period or loss to follow-up. There were no missing data for the current analysis.

### Endpoint

The primary endpoint was the absolute reduction in the rate of severe exacerbations with mepolizumab 75 mg compared to placebo in the first 365 days of follow-up. In line with the American Thoracic Society (ATS) / European Respiratory Society (ERS) Task Force definition [26], a severe exacerbation was defined as a worsening of asthma which required OCS for at least 3 days, hospitalization, or emergency department (ED) visit requiring systemic corticosteroids. The secondary endpoint was reduction in the 5-item Asthma Control Questionnaire (ACQ5) score with mepolizumab 75 mg compared to placebo over the first 365 days of follow-up [27]. The ACQ5 score integrated five questions on asthma symptom control into a 0 (excellent control) to 6 (extremely poor control) scale.

### Covariates of interest

Consistent with a previous analysis on the heterogeneity in the burden of exacerbations in eosinophilic asthma [28], this analysis focused on 15 commonly recorded patient characteristics, including age, sex (male or female), ethnicity (Hispanic or other), body mass index (BMI), history of smoking (yes or no), duration of asthma, pre-bronchodilator value of Forced Expiratory Volume in 1 s (FEV_1_), FEV_1_ bronchodilator responsiveness (in %), the ratio of post-bronchodilator FEV_1_/Forced Vital Capacity (FVC), a history of nasal polyps, BEC, serum IgE level, long-term OCS use, ACQ5 score, and number of previous severe exacerbations, all measured at baseline or the 12 months preceding study enrolment. Of note, fractional exhaled nitric oxide (FeNO) was not included because it was measured only in DREAM but not in MENSA.

### Statistical analysis

All statistical analyses were performed in R (version 3.6.0, 2020). P-values were considered significant at two-tailed 0.05 level. Descriptive statistics were compared using Pearson Chi-square test for categorical variables and Kruskal–Wallis test for continuous variables.

To construct the treatment benefit prediction model for the primary endpoint (exacerbation rate reduction), we fitted a generalized linear model (GLM) with Poisson distribution and logarithmic link function, with the number of severe exacerbations during follow-up as the dependent variable and the logarithm of total follow-up days as the offset. The covariates and their first-order interaction with treatment were the independent variables. To prevent overfitting, we applied Lasso regression to estimate model parameters, which shrank the coefficients of non-essential covariates to zero [29]. We used 10-fold cross validation to identify the optimal shrinkage factor that minimized the mean squared error of off-sample predictions. For the secondary endpoint (reduction in ACQ5 score), we fitted a Lasso GLM model with normal distribution and logarithmic link function, with the last follow-up ACQ5 score as the dependent variable. Variable specification, selection, and model fitting steps were the same as for the primary endpoint, except the total days of follow-up was included as an independent variable (assuming a log-linear effect for time since randomization on the ACQ5 score).

We predicted treatment response for each individual, based on the fitted GLM models, as the difference in the predicted rate of exacerbation or the ACQ5 score between treatment and no treatment [30].

To evaluate predictive performance for both endpoints, we assessed model calibration based on calibration plots and the root mean squared error, with details provided in *Appendix S2*. Because this study aimed to quantify the predictive capacity of patient characteristics, rather than deriving a final prediction tool, external validation was not assessed.

The Lasso-selected predictors were ranked based on their importance in predicting treatment response. Importance was quantified as the absolute value of magnitudes of coefficients in a linear regression model with predicted treatment response as the dependent variable and standardized predictors as independent variables.

### Assessment of the heterogeneity of treatment benefit

A successful individualized prediction algorithm should be able to identify the subgroup of individuals who benefit the most from treatment, or those who benefit the least [23]. As such, measures of concentration of a variable within a population, in particular the Lorenz curve and the closely related Gini index, can be relevant to this context [31]. The more concave the Lorenz curve, the higher the capacity of covariates in concentrating treatment benefit. The Gini index is a summary statistic of inequality, which is 0 with perfect equality and with higher values indicating higher levels of inequality [31]. The Gini index is widely used to measure inequality in the distribution of economic resources [32], and has also been applied to national and international data to quantify health inequalities [33–35]. If salient patient characteristics are capable of distinguishing individuals who will benefit the most from treatment, then prioritizing treatment for subgroups of patients with higher predicted benefit should be significantly more efficient than treating patients indifferently (thus informing treatment rule-in decisions). Similarly, a powerful treatment benefit prediction algorithm should be able to identify patients who will not benefit from therapy (thus informing treatment rule-out decisions). To explore the capacity of covariates to inform treatment rule-in or rule-out, we assessed how the average treatment benefit would change if mepolizumab were given to quintiles of patients with higher (or lower) predicted benefits. The observed treatment benefit for the primary endpoint was defined as the average difference in the annualized rates of severe exacerbation between the placebo and treatment arms; the observed treatment benefit for the secondary endpoint was defined as the average reduction in ACQ5 in the last follow-up visit between the treatment and placebo arms.

## Results

Table 1 presents the baseline characteristics of the study cohort. The placebo group consisted of 320 patients (mean age 47.6 years, 60% female, median follow-up 237 days); the treatment group consisted of 314 patients (mean age 49.8 years, 60% female, median follow-up 235 days).

**Table 1:** Baseline characteristics of the analytical sample

Selected Predictors	Placebo (n = 320)	75 mg Mepolizumab (n = 314)	p-value
**Age (years), mean (SD)**	47.6 (13.3)	49.8 (13.1)	0.027
**Female, n (%)**	191 (59.7)	192 (61.1)	0.71
**Ethnicity (Hispanic or Latino), n (%)**	31 (9.7)	32 (10.2)	0.83
**Smokers, n (%)**	67 (20.9)	74 (23.6)	0.43
**Body Mass Index (kg/m** ^**2**^ **), mean (SD)**	28.2 (5.9)	27.9 (5.8)	0.80
**Duration of asthma (years), mean (SD)**	18.8 (14.3)	19.1 (13.8)	0.55
**Nasal polyps, n (%)**	45 (14.1)	40 (12.7)	0.62
**Percentage of predicted pre-bronchodilator FEV**_**1**_, **mean (SD)**	61.0 (17.0)	60.3 (17.5)	0.75
**FEV**_**1**_ **Reversibility (%), mean (SD)**	26.8 (22.4)	26.3 (20.3)	0.92
**Post-bronchodilator FEV**_**1**_ **/FVC, mean (SD)**	0.65 (0.13)	0.66 (0.13)	0.43
**ACQ5 score, mean (SD)**	2.33 (1.12)	2.17 (1.10)	0.10
**Blood eosinophil count (×10** ^**9**^ **/L), mean (SD)**	0.44 (0.39)	0.41 (0.38)	0.16
**Serum IgE (U/ml), mean (SD)**	434.3 (853.4)	552.0 (1323.7)	0.50
**Maintenance daily dose of oral corticosteroid, n (%)**	79 (24.7)	79 (25.2)	0.89
**No. of severe exacerbations in the previous year, mean (SD)**	3.6 (3.4)	3.6 (2.7)	0.64
**No. of exacerbations requiring hospitalization in previous year, mean (SD)**	0.6 (1.5)	0.5 (1.0)	0.75

ACQ5, 5-item Asthma Control Questionnaire, FEV_1_, forced expiratory volume at 1 s; FVC, forced vital capacity, SD, standard deviation. IgE: immunoglobulin E

### Prediction of treatment benefit and predictor importance

The predicted severe exacerbation rate was 0.71/year lower in the treatment versus the placebo group with 365 days of treatment (average annual rate of exacerbation: 1.13/year in the mepolizumab group vs. 1.84/year in the control group). The predicted difference in ACQ5 score over 365 days was 0.22 lower in the in the treatment versus the placebo group (average ACQ5 score: 1.40 in the exposure group vs. 1.62 in the control group) (*Appendix Figure*S1 for the distributions of predicted treatment benefit). Of note, a change of 0.5 in the ACQ5 score is considered to be minimally clinically significant [36].

**Fig. 1:**
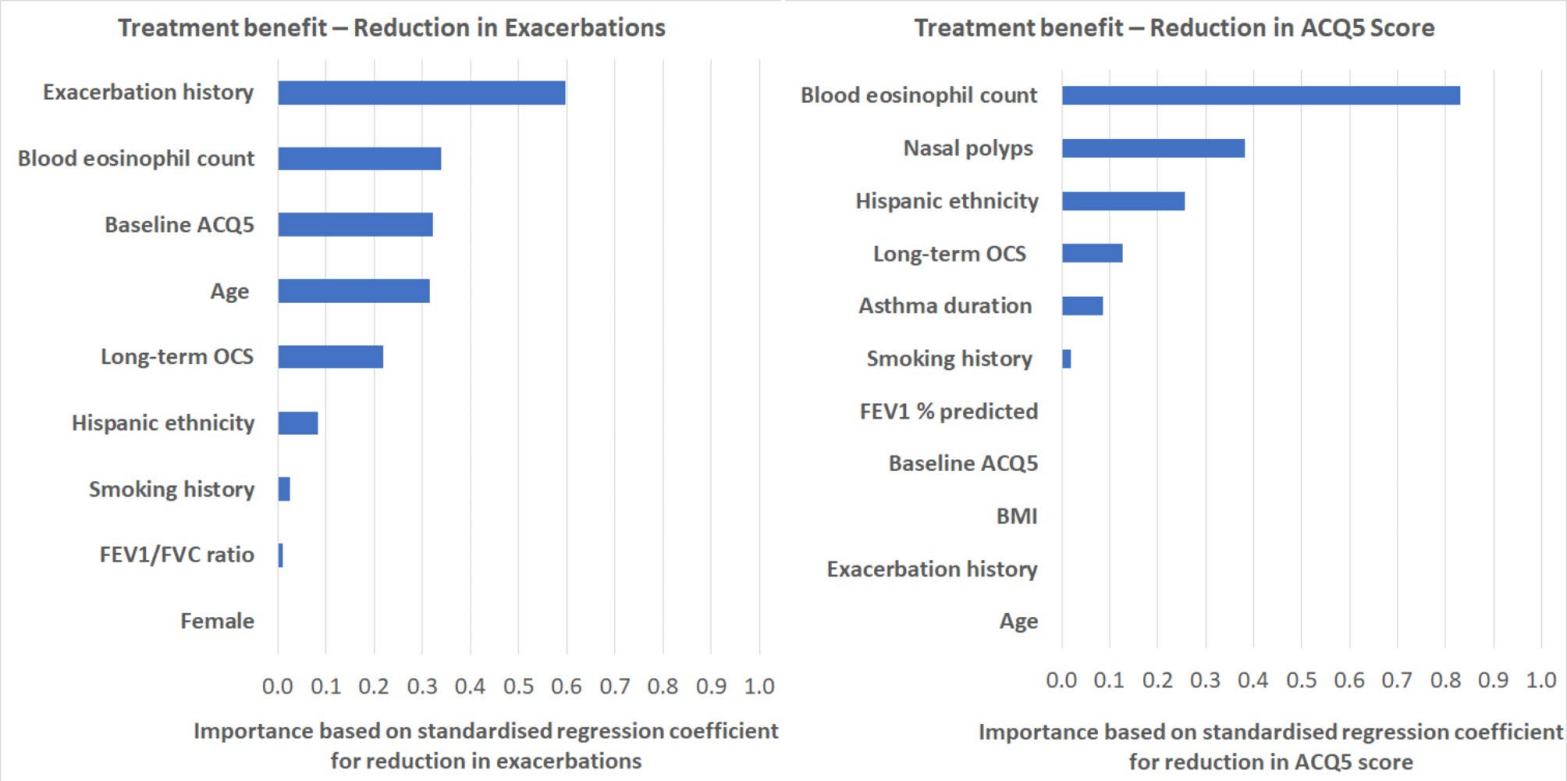
Importance of individual standardized covariates for predicting the reduction of severe exacerbations and the 5-item Asthma Control Questionnaire (ACQ5) score with 1-year mepolizumab treatment. Left panel, reduction in rate of severe exacerbations in 365 days of follow up. Right panel, reduction in ACQ5 scores in 365 days of follow up. The x-axis shows the absolute value of the regression coefficient for each covariate. BMI, body mass index, FEV_1,_ forced expiratory volume at 1 s, FVC, forced vital capacity, OCS, oral corticosteroids

The model calibration results are reported in *Appendix S2*, demonstrating good calibration for both outcomes. Figure 1 shows the ranking of predictor importance to treatment response. Exacerbation history, baseline BEC, baseline ACQ5 score, baseline age, as well as long-term OCS use were the major predictors of treatment benefit for severe exacerbations, while baseline BEC, the presence of nasal polyps, ethnicity, and long-term OCS use were the major predictors of treatment benefit for symptom control.

### Heterogeneity of treatment benefit

**Fig. 2:**
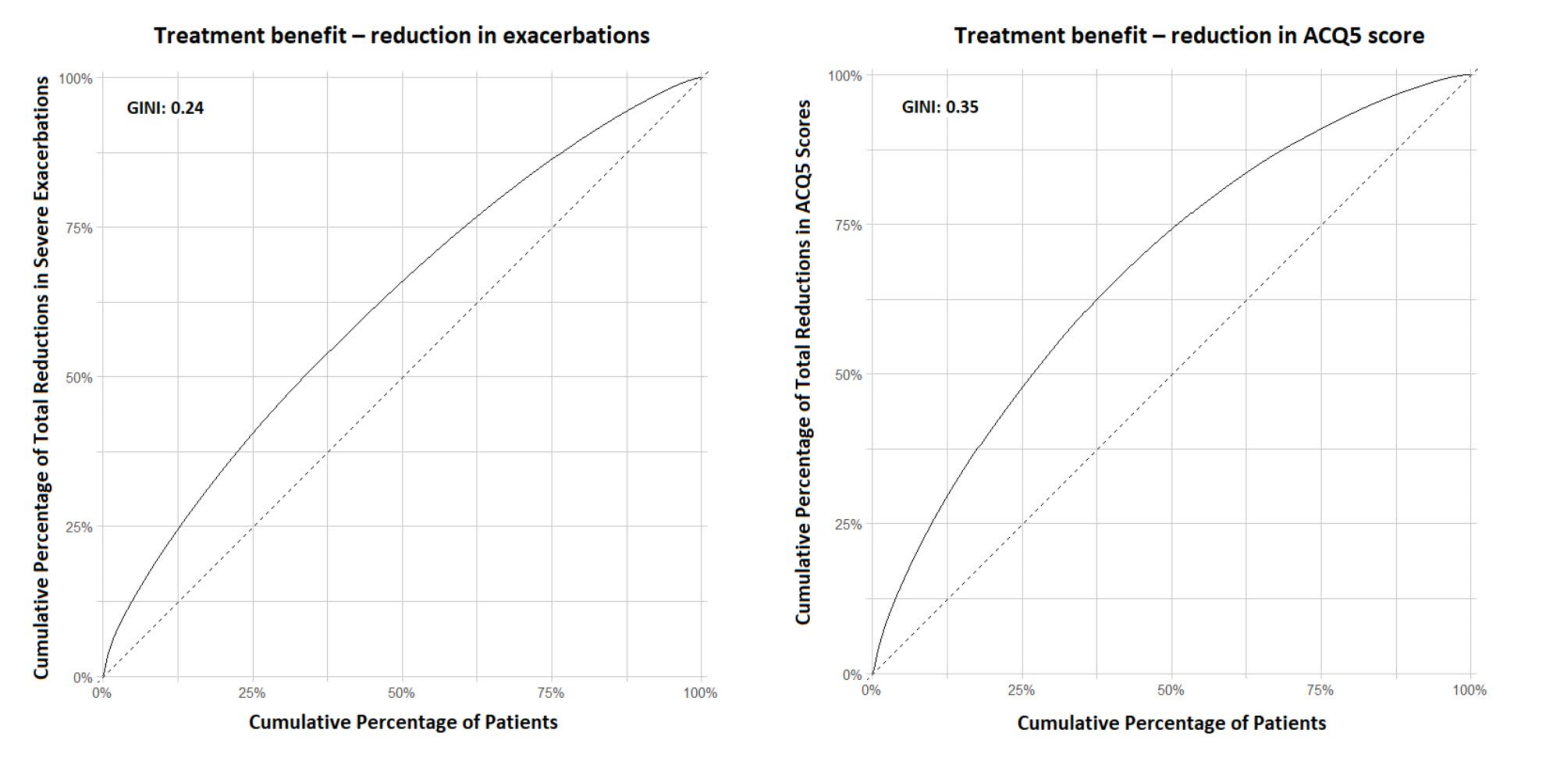
Lorenz curve for the heterogeneity of treatment benefit comparing 75 mg mepolizumab versus placebo in severe asthma. Left panel, predicted reduction in rate of severe exacerbations in 365 days of follow up. Right panel, predicted reduction in ACQ5 scores in 365 days of follow up. The Gini Index captures the level of inequality in predicted treatment benefit

Figure 2 shows the Lorenz curves for the reduction in severe exacerbation rate and improvement in symptom control associated with mepolizumab treatment. The Lorenz curves were both above the line of equality (45-degree line), suggesting the presence of heterogeneities in predicted treatment benefits in both domains. The Lorenz curve was more concave for symptom control (ACQ5 score) than for exacerbations. For instance, if mepolizumab were to be given to all patients, the 50% of patients with the highest predicted treatment benefit would experience 66% of total reductions in severe exacerbations, and 75% of total reductions in ACQ5 score. The Gini Index was 0.240 (95% CI, 0.221, 0.268) for the reduction of exacerbations and 0.348 (95% CI, 0.330, 0.372) for the reduction of ACQ5 score.

**Fig. 3:**
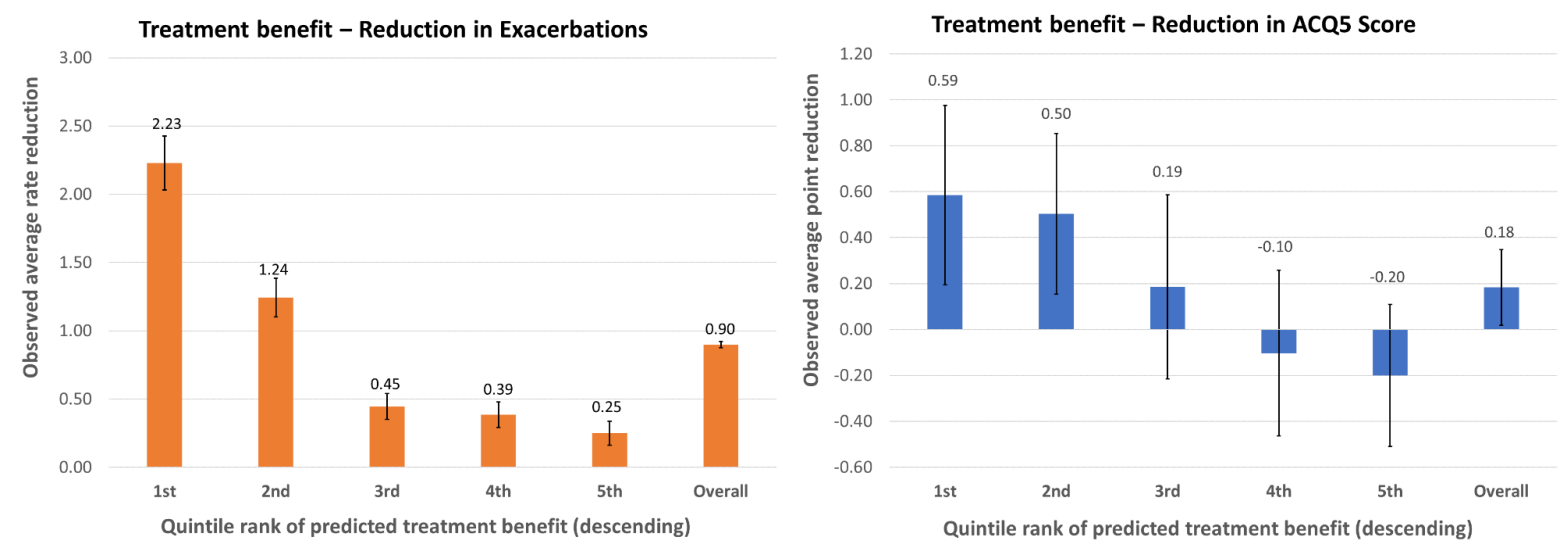
Averaged treatment effect comparing mepolizumab 75 mg versus placebo in severe asthma across quintiles of predicted treatment benefit. Left panel, observed treatment benefit in terms of average reduction in the rate of severe exacerbations per year; Right panel, observed treatment benefit in terms of average reduction in ACQ5 scores

Figure 3 displays the observed treatment benefit for severe exacerbation rate and ACQ5 score, by quintiles of predicted treatment benefit. Overall, the predicted treatment benefit for average reduction in exacerbations was 0.90/year (95%CI, 0.87‒0.92) and that for average reduction in ACQ5 score was 0.18 (95% CI, 0.02‒0.35). Consistent with findings based on the Gini index, patient characteristics in combination were better predictors of improvement in symptom control compared to reduction of exacerbations. For example, comparing to treating all patients, targeting the top 20% of patients with the highest predicted benefit for exacerbations would increase the observed benefit (i.e., average absolute reduction in observed rate of severe exacerbations) from 0.90/year (95%CI, 0.87‒0.92) to 2.23/year (95% CI, 2.03‒2.43), corresponding to a 248% increase in treatment efficiency. Meanwhile, targeting the 20% of patients with the highest predicted benefit for asthma control would change the observed treatment benefit (i.e., average reduction in ACQ5 score in the last visit) from 0.18 (95% CI, 0.02‒0.35) to 0.59 (95% CI, 0.19‒0.98), i.e., 328% improvement in treatment efficiency. Furthermore, patient characteristics were even more effective in identifying subgroups with a low rate of response for both exacerbation and asthma control. For instance, comparing to treating all patients, targeting the 20% of patients with the lowest predicted benefits for exacerbations would reduce the observed treatment benefit from 0.90/year to 0.25/year (95% CI, 0.16‒0.34), i.e., 72% reduction, and for asthma control from 0.18 to -0.20 (95% CI, -0.51 to 0.11).

Of note, covariates in combination outperformed the capacity of any single predictor. For example, consider BEC as a single biomarker to predict treatment benefit, which was a most important predictor for treatment response to both exacerbation and symptom control (Fig. 1). The BEC model was associated with a Gini Index of 0.106 (95% CI, 0.098, 0.120) for the reduction of exacerbations and nearly 0 for the reduction of ACQ5 score, while the Gini Index of the full model was respectively 0.240 and 0.348. Thus, BEC alone was much less able to identify sub-groups with differential responses compared to that predicted from combined patient characteristics.

## Discussion

We evaluated the combined capacity of salient patient characteristics in predicting the benefit of mepolizumab in reducing severe exacerbation rates and improving asthma symptom control. We quantified their capacity in terms of disparities in treatment response via the Lorenz curve and the Gini index, and in terms of how the observed treatment benefit changed over quintiles of predicted benefit. Patient characteristics captured higher heterogeneity in individual treatment response for improving asthma control than for exacerbation reduction (Gini indices of 0.35 vs. 0.24). Similarly, treatment benefit varied more widely across quintiles of predicted benefit for asthma control than for exacerbation rate. As well, patient characteristics showed greater potential for predicting below-average than above-average treatment response. On the other hand, while patient characteristics could identify subgroups with low treatment response, they did not identify any subgroup that would be harmed by treatment (while observed treatment benefit was negative in the bottom two quintiles for ACQ5 score, it did not achieve statistical significance).

How do these results inform the development of precision medicine for severe asthma? This was a feasibility study, not ready for dissemination as a clinical prediction model. Such an algorithm would require a more formal model development and external validation approach. However, before embarking on such a process, it is beneficial to know whether patient characteristics can indeed explain heterogeneity in treatment effect. Our results show that a precision medicine approach based on clinical prediction modeling could indeed result in more efficient treatment assignment rules, even among patients who have satisfied the strict eligibility criteria of severe asthma trials. This finding can have important implications. A recent systematic review concluded that the prices of biologics would need to be reduced by at least 60% to meet the threshold for cost-effectiveness [37]. An alternative way to improve the value-for-money potential of biologics is to enable ‘concentration of benefit’ by adopting a stratified treatment algorithm that prioritizes treatment to individuals with high likelihood of treatment response. For instance, based on our results, a 60% improvement in the efficiency of mepolizumab would be achieved in terms of asthma symptom control if it were given to the 40% of eligible patients with the highest likelihood of improvement in symptom control, or to the 20% of eligible patients with the highest predicted reduction in exacerbations. These results were obtained despite the fact that patients were already pre-selected for the likelihood of response to biologics, as reflected in the inclusion criteria of the two RCTs. Our findings therefore indicate that a more accurate selection for treatment based on a combination of patient characteristics is feasible. Such gain in efficiency of treatment decisions is unlikely to be achieved by the use of a single biomarker. This was demonstrated by the significantly better performance of the combined set of patient characteristics over BEC alone, which is an important component of biologic eligibility criteria [38]. Further, the finding that patient characteristics were more capable of identifying below-average compared with above-average response suggest a potentially high utility of prediction models for ruling out treatment, or otherwise identifying patients who might require more intense monitoring and evaluation due to lower likelihood of treatment response. Finally, the importance ranking of predictors from our study can inform the selection of predictors or the development of clinical prediction models for treatment response.

To the best of our knowledge, this study is the first to quantify the combined capacity of patient characteristics towards predicting treatment response to a biologic in patients with severe asthma. Use of standardized clinical measurements and robust statistical analyses enhance the validity of our findings. This study also has several limitations. First, the generalizability and applicability of the current findings require careful consideration because the clinical trial data were limited by the narrow eligibility criteria of the RCTs (already selecting patients for high likelihood of response) and relatively small sample size. In addition, exacerbation rate and asthma control in the comparison group might be biased estimates of their counterparts in the general severe asthma population due to a placebo effect of participating in a clinical trial [39]. However, real-world prescribing practice often deviates from guidelines for use of biologic therapies, which may also introduce bias to the prediction affecting the generalizability of our findings. For instance, a US-based claims data analysis showed that the majority of asthma patients initiating biologic therapy did not have severe asthma and many were poorly adherent to first-line asthma treatment, indicating inappropriate treatment escalation [40]. Therefore, external validation of the development of clinical prediction models should be performed in real-world data with adjustment for medication adherence and other real-world factors for biologic initiation [41], which should include a broader class of biologic drugs. Further, our analysis was performed with only one biologic (i.e., mepolizumab) at a single intravenous dose (75 mg) which was available in both MENSA and DREAM, whereas the effect of biologics may vary across type, dosage, and patient population. The more widely used 100-mg subcutaneous mepolizumab was available only in MENSA which could not produce a sufficient sample size to perform the desired shrinkage [25]. Nonetheless, improvements in asthma exacerbation and other outcomes such as FEV1 were similar between these two doses and modes of delivery in MENSA [10]. Third, other domains of treatment response, such as reduction in OCS exposure and improvement in quality of life, were not explored in this analysis. Last but not least, other important risk factors such as FeNO level, comorbidities, atopy status, socioeconomic status, environmental exposures, and other medication use were not recorded in the data and thus their predictive capacity could not be explored. On the other hand, several prognostic factors such as biomarkers and lung function parameters are not routinely collected in clinical practice, particularly in primary care, which could limit the applicability of a full prediction model in the future.

## Conclusion

While no single patient characteristic was a strong predictor for benefit of mepolizumab in severe asthma, the combination of multiple characteristics could reasonably predict treatment response of mepolizumab in severe asthma, in particular improvement in asthma symptom control. This indicates that individualized, covariate-informed treatment rules have the potential to improve the efficiency of biologic therapies and their value proposition. Such gain in efficiency should be pursued in tandem with other efforts in improving the efficiency of biologic therapy, such as ensuring the treatment is given to truly eligible patients, improving adherence to inhaled therapies and inhaler technique before prescribing biologics, and continuing negotiations on the price of biologics. These findings call for future research on developing precision medicine tools for treatment selection and validating them in representative samples.

## Electronic supplementary material

Below is the link to the electronic supplementary material.

Supplementary Material 1

## Data Availability

The data that support the findings of this study are available from GlaxoSmithKline but restrictions apply to the availability of these data, which were used under license for the current study, and so are not publicly available. Data are however available from the authors upon reasonable request and with permission of GlaxoSmithKline.

## Abbreviations

ACQ5: 5-item Asthma Control Questionnaire
ATE: average treatment effect
ATS: American Thoracic Society
BEC: blood eosinophil count
BMI: body mass index
DREAM: the Dose Ranging Efficacy And safety with Mepolizumab
ED: emergency department
ERS: European Respiratory Society
FEV_1_: forced expiratory volume in 1 s
FVC: forced vital capacity
GLM: generalized linear model
MENSA: the Mepolizumab as Adjunctive Therapy in Patients with Severe Asthma
NNT: numbers needed to treat
RCT: randomized controlled trials
OCS: oral corticosteroids
