# Supplementary material for: Can we predict who will benefit most from biologics in severe asthma? A post-hoc analysis of two phase 3 trials
Source: Respir Res. 2023 May 2;24:120. doi: 10.1186/s12931-023-02409-2 (PMC10155396; doi:10.1186/s12931-023-02409-2)

# Can we predict who will benefit most from biologics in severe asthma? A post-hoc analysis of two phase 3 trials

Wenjia Chen, PhD; Helen K. Reddel, MBBS, PhD; J Mark FitzGerald, MD; Richard Beasley, DSc; Christer Janson, MD, PhD; Mohsen Sadatsafavi, MD, PhD

# Appendix S1. Inclusion criteria of DREAM and MENSA

During the 52-week DREAM study, 621 patients were randomly assigned (in a 1:1:1:1 ratio) to receive one of the three doses of intravenous mepolizumab (75mg, 250mg, 750mg) or matched placebo every 4 weeks. During the 38-week MENSA study, 576 patients were randomized (in a 1:1:1 ratio) to receive placebo, or a 75mg intravenous dose or a 100mg subcutaneous dose of mepolizumab, every 4 weeks. Both studies had similar inclusion criteria: patients aged 12 years and above, a clinical diagnosis of asthma by a physician, and pre-bronchodilator forced expiratory volume at 1 second (FEV_1_) of less than 80% of the predicted value, or variability in diurnal peak expiratory flow of more than 20% during run-in. Further, these patients were required to have eosinophilic asthma or a high likelihood of eosinophilic asthma as indicated by a blood eosinophil count of ≥300/μL in DREAM or ≥150/μL in MENSA, a sputum eosinophil count of ≥3% in DREAM, and/or an exhaled nitric oxide concentration (FeNO) of ≥50 ppb in DREAM within in the previous 12 months. In addition, both studies required patients to have a confirmed history of 2 or more asthma exacerbations requiring treatment with oral corticosteroids (OCS) in the preceding 12 months.

# Appendix S2. Model calibration

Model calibration was assessed in the following ways. First, a calibration plot was drawn to visualize the agreement between observed outcomes and predictions across deciles. Second, the root mean squared error (RMSE) was calculated, which is directly related to the standard deviation of prediction error (i.e., residuals) and in the same unit as the outcome. Because this study aimed to quantify the predictive capacity and identify effect modifiers rather than deriving a final prediction tool, external validation was not assessed.

Lasso models starting with the same set of salient risk factors and treatment-by-covariate interaction terms had a RMSE of 1.52 in prediction of exacerbations and 0.93 in prediction of ACQ5 score. The two calibration plots below show that, the salient patient and disease characteristics performed slightly better in predicting ACQ5 scores than rates of severe exacerbations.

Appendix S2 Figure A). Calibration plot for prediction of severe exacerbations in 365 days of follow-up. Line, prediction; Point estimates with error bars, observed value ± standard deviation.


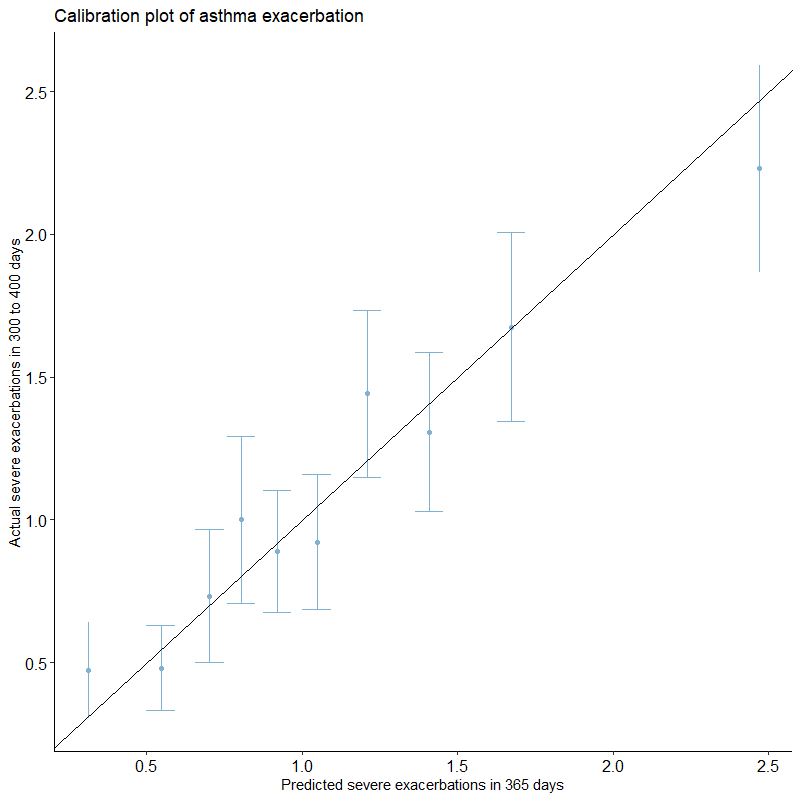


Appendix S2 Figure B). Calibration plot for prediction of ACQ5 score in 365 days of follow up. Line, prediction; Point estimates with error bars, observed value ± standard deviation.


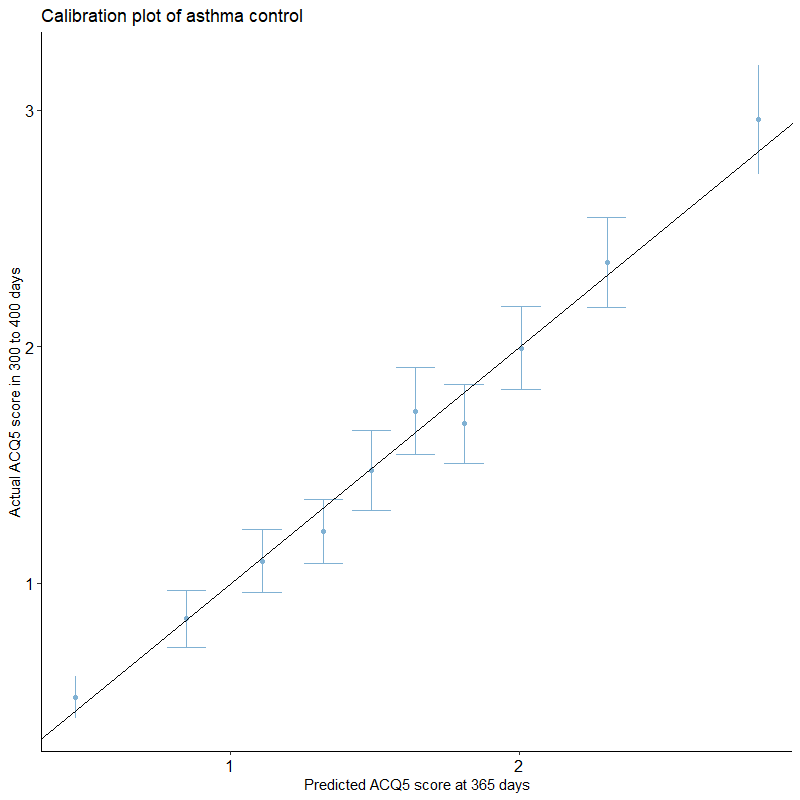


# Appendix Figure S1. Histogram of expected treatment benefits with 1-year 75mg mepolizumab.

A) Predicted treatment benefit in terms of the reduced numbers for severe exacerbations over 365 days of follow up.


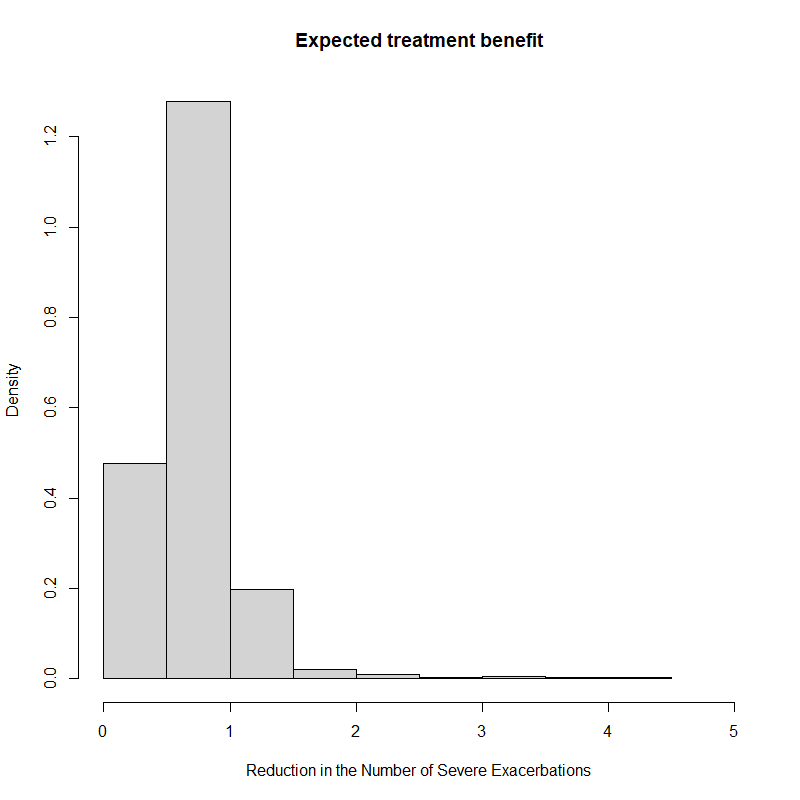


B) Predicted treatment benefit in terms of the reduction in ACQ5 scores over 365 days of follow up.


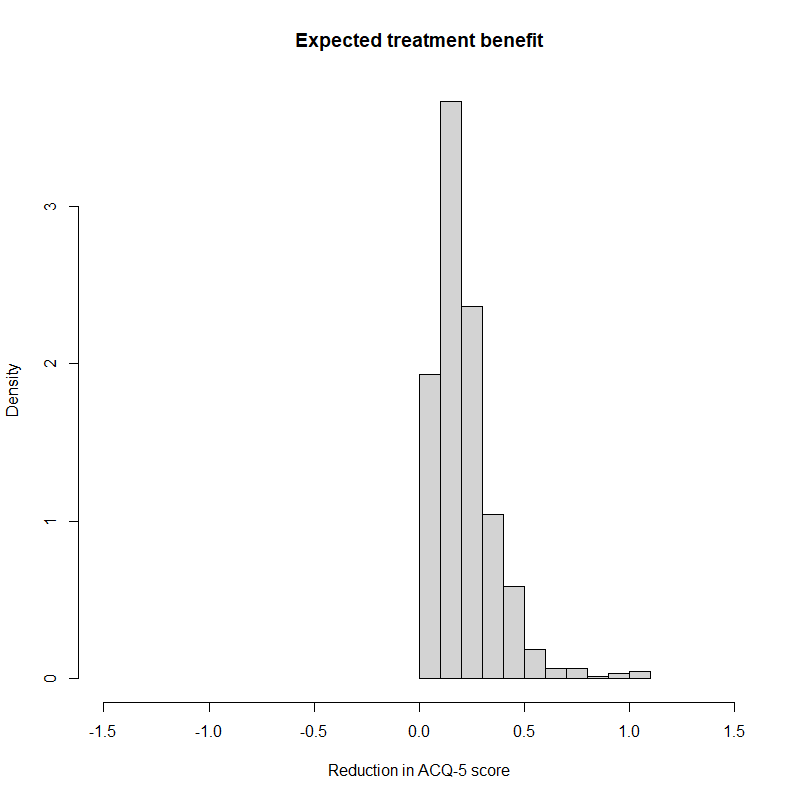

Supplement: Supplementary file 1 — Supplementary Material 1 [file 12931_2023_2409_MOESM1_ESM.docx]
